# Fundamental basis of patient-specific caries prevention

**DOI:** 10.1186/1878-5085-5-S1-A118

**Published:** 2014-02-11

**Authors:** Dmitry A Kunin

**Affiliations:** 1 Voronezh N.N. Burdenko State Medical Academy, Dentistry Department, Voronezh, Russia

## 

Clinical introduction of methods for personalized preventive medicine, it is essential to unravel biochemical components and ultastructural features of hard dental tissues. Accordingly, utilizing a variety of techniques and protocols (e.g., electron probe microanalysis, atomic-powered tunnel electron microscopy), the essential structural features of enamel (e.g., “enamel bridges” and “enamel tunnels”) have been identified. These studies have also led to our better understanding of some metabolic processes in teeth.

Detection of previously unrecognized structures in enamel and dentin has helped explain the selectivity of chemical elements and their actions in the context of preventive means and the necessity of personalization of carious and not carious teeth diseases prevention.

Our studies suggest that “enamel tunnels” play a major role in exchange processes between the tooth enamel and oral fluid stimulating mineral penetration of the dental enamel by molecular substances and microorganisms (i.e. functioning as a tissue barrier). Therefore, the enamel surface must be kept unexposed and the tooth openings must be treated to prevent its morphochemical alterations. The protective zone is non-cariogenic dental plaque sublimating necessary organic and inorganic components of the oral fluid. It appears difficult to accumulate this “plaque” but it becomes possible with the preventive means. Therefore, it is imperative to improve the professional oral care techniques in order to determine an acceptable level of caries prevention efficiency.

